# Combination of HIV-1 and Diabetes Enhances Blood Brain Barrier Injury via Effects on Brain Endothelium and Pericytes

**DOI:** 10.3390/ijms21134663

**Published:** 2020-06-30

**Authors:** Slava Rom, Sachin Gajghate, Malika Winfield, Nancy L. Reichenbach, Yuri Persidsky

**Affiliations:** 1 Department of Pathology and Laboratory Medicine, Lewis Katz School of Medicine, Temple University, Philadelphia, PA 19140, USA; gajghate@temple.edu (S.G.); malika.winfield@temple.edu (M.W.); nancylee@temple.edu (N.L.R.); 2 Center for Substance Abuse Research, Lewis Katz School of Medicine, Temple University, Philadelphia, PA 19140, USA

**Keywords:** BBB, diabetes, HIV-1, hyperglycemia, endothelial dysfunction, actin cytoskeleton rearrangement, pericytes

## Abstract

Despite combined antiretroviral therapy (ART) achieving efficient HIV replication control, HIV-associated neurocognitive disorders (HAND) continue to be highly prevalent in HIV-infected patients. Diabetes mellitus (DM) is a well-known comorbidity of HAND in HIV-infected patients. Blood brain barrier (BBB) dysfunction has been linked recently to dementia development, specifically in DM patients. BBB injury exists both in HIV and DM, likely contributing to cognitive decline. However, its extent, exact cellular targets and mechanisms are largely unknown. In this report, we found a decrease in pericyte coverage and expression of tight junction proteins in human brain tissues from HIV patients with DM and evidence of HAND when compared to HIV-infected patients without DM or seronegative DM patients. Using our in vitro BBB models, we demonstrated diminution of barrier integrity, enhanced monocyte adhesion, changes in cytoskeleton and overexpression of adhesion molecules in primary human brain endothelial cells or human brain pericytes after exposure to HIV and DM-relevant stimuli. Our study demonstrates for the first-time evidence of impaired BBB function in HIV-DM patients and shows potential mechanisms leading to it in brain endothelium and pericytes that may result in poorer cognitive performance compared to individuals without HIV and DM.

## 1. Introduction

Cognitive impairment/dementia progression and its associations with diverse pathologic conditions remain mysterious. A number of fundamental mechanisms/causes have been suggested including genetic defects in Parkinson’s disease and Alzheimer’s disease (AD), hypertension, metabolic abnormalities (diabetes mellitus (DM), homocysteinemia, toxic effects of drugs and environmental factors, etc.), chronic neuroinflammatory conditions (autoimmune or infectious origin), stroke and traumatic brain injury. Nevertheless, close to 40% of these ailments converge on defects in the brain microvasculature [1] and its coupling to neuronal functions, causing cerebral hypo-perfusion and subsequent neuronal failure [2,3,4].

Human immunodeficiency virus (HIV)-1 crosses the blood brain barrier (BBB) and infects microglial cells in the central nervous system (CNS). HIV-1-associated neurological disorder (HAND) is described as cognitive, motor and/or behavioral deficiencies initiated by HIV replication, immune activation and neurotoxin secretion in the brain that result in neuronal damage [5]. Despite antiretroviral therapy during which some individuals display immune recovery, the incidence of HAND remains high, and its causes are poorly understood [5]. One current theory is that HIV-1-associated neurodegeneration is forced by chronic inflammatory processes in the brain, secondary to a low level of HIV-1 replication in CNS reservoir cells (macrophages, microglia) [6,7].

Recently, cognitive demise has been described in patients suffering from DM type 1 or type 2 [8,9,10]. DM is a metabolic disease associated with the failure to produce insulin and appropriate utilization of glucose (type 1) or insulin resistance when this hormone is produced but unable interact with its receptors (type 2). Both DM conditions result in high glucose levels in blood, increased production of reactive oxygen species (ROS) and end organ injury stemming from microvascular abnormalities. Cardiovascular disease, nephropathy and retinopathy secondary to microvascular injury are well-documented DM complications [11]. The association between microvascular changes and cognitive decline in DM has not been validated until very recently, including impaired blood flow, neuronal dysfunction, abnormalities of white matter microstructure and metabolic aberrations [12]. Overall small vessel abnormalities documented in DM include white matter lesions, microbleeds, silent brain infarcts and lacunar abnormalities indicative of cognitive decline [13].

BBB chronic demise and its association with cognitive decline has been recently found in DM patients [14]. Cognitive decline in DM patients was associated with increased levels of vascular dysfunction markers in cerebrospinal fluid (CSF) [14]. Markers of BBB damage have better predictive power for dementia development than genetic hallmarks of AD. Further, memory deficits have been documented in animal models of DM, which showed BBB dysfunction paralleling a loss of pericytes and neuronal dysfunction [4,15,16]. Recently, we showed an increased BBB permeability that was associated with hyperglycemia in both DM types 1 and 2 animal models [3,4]. Notably, these animals revealed cognitive decline paralleling BBB injury. Profiling of genes in CNS microvessels isolated from these animals presented upregulation of pro-inflammatory and barrier-destabilizing molecules, and the extent of their expression interrelated with cognitive defects.

Recent publications have suggested a strong link between DM and HAND [17,18]. Further, patients with DM and HIV performed significantly worse on psychomotor tests when compared to HIV patients without DM [19]. Our previous study indicated BBB injury enhanced neuroinflammation and decreased pericyte microvessel coverage in human brain tissues derived from HIV-infected patients (with or without antiretroviral therapy (ART)) [20]. DM is a well-known correlate of HAND in HIV-infected patients [21,22]. The role of comorbidities in HAND development is one of the top research priorities according to the NIMH recent workshop “NeuroHIV in the ART era” [23].

In the present study, we demonstrated that the combination of DM conditions and presence of HIV-1_ADA_ resulted in diminished BBB tightness, which concurred with increased expression of plasmalemma vesicle associated protein (PLVAP), a marker of brain endothelial cell permeability [24,25,26] and actin cytoskeletal rearrangements. Pericyte cells supporting BBB function also displayed increased cytoskeletal actin reorganizations and the expression of the crucial pericyte receptor, PDGF-Rβ, which assures proper pericyte function and barrier integrity [27] was decreased. This report offers evidence from in vitro BBB models and DM HIV patients for the causes of vascular dementia and might lead to development of future therapeutics to reduce its burden.

## 2. Results

### 2.1. HIV Infection and Hyperglycemia Result in Decreased Claudin-5, TJ Protein, and CD13 Expression in Cerebral Microvessels of DM Patients with HIV

**Figure 1:**
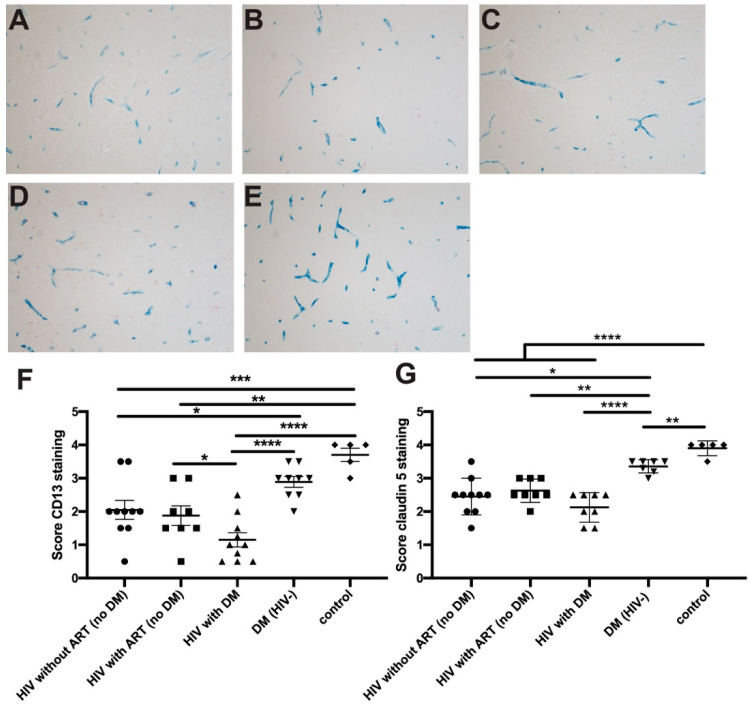
Decrease of claudin-5 expression and pericyte coverage of BBB (blood brain barrier) in DM (diabetes mellitus), HIV-infected and HIV patients with DM. (**A**,**B**) There was a significant decrease in CD13 staining in HIV-infected patients with or without ART (antiretroviral therapy). (**C**) CD13 was further diminished in HIV-DM patients when compared to DM seronegative patients (**D**) or controls (**E**). All images were acquired with 20 ms exposure. Original magnification: A-E x200. Semiquantitative assessment of CD13 (**F**) and claudin-5 expression (**G**) in HIV-infected patients without ART (*n* = 10), HIV-ART (*n* = 11), HIV-DM (*n* = 11), DM without HIV (*n* = 11) or control subjects (*n* = 5). Group mean and standard error are indicated by bars. * *p* < 0.05; ** *p* < 0.01; *** *p* < 0.001; **** *p* < 0.0001.

To assess the effects of DM and HIV on cerebral microvasculature, we tested expression levels of the TJ protein, claudin-5 and of the pericyte markers, PDGF-Rβ and CD13, in cerebral microvessels. Semi-quantitative analysis showed that there was a significant decrease in CD13 staining in HIV-infected patients (with *p* = 0.002 or without ART *p* = 0.001, Figure 1A,B) when compared to seronegative controls (Figure 1E,F). These changes were even more obvious in HIV-infected patients with DM where CD13 expression was significantly lower when compared with HIV patients (without ART), DM HIV-negative (*p* = 0.02, Figure 1D) or controls (*p* < 0.0001, Figure 1E). Of note, the pericyte marker was significantly diminished in HIV-negative DM patients as compared to controls (*p* < 0.001, Figure 1F). As in our prior study, diminished expression of pericyte markers was not different between ART-treated or untreated patients. We also found significantly attenuated staining for claudin-5 in HIV-infected patients as compared to controls Figure 1G). Further decrease in claudin-5 staining was seen in HIV patients with DM Figure 1G). There was a notable attenuation of claudin-5 staining in human brain tissues from seronegative patients with diabetes and HIV-infected patients especially evident in patients with DM and HIV (Figure 1G and Supplemental Figure S1A,C,E,G). Decline in claudin-5 staining paralleled diminution of PDGF-Rβ staining (pericyte markers) (Supplemental Figure S1B,D,F,H). HIV-driven chronic inflammation leads to decreases in TJ protein expression, resulting in BBB injury [28,29]. Disruption of the BBB and its association with cognitive decline has been recently demonstrated in DM patients [14] and DM mouse models [3,4]. Therefore, barrier compromise exists in DM and HIV infection, and it is aggravated by a combination of both due to the same cellular and molecular targets.

### 2.2. Hyperglycemia and HIV Exposure Diminish BBB Tightness and Intensify Primary Human Monocyte Adhesion to BMVEC

**Figure 2:**
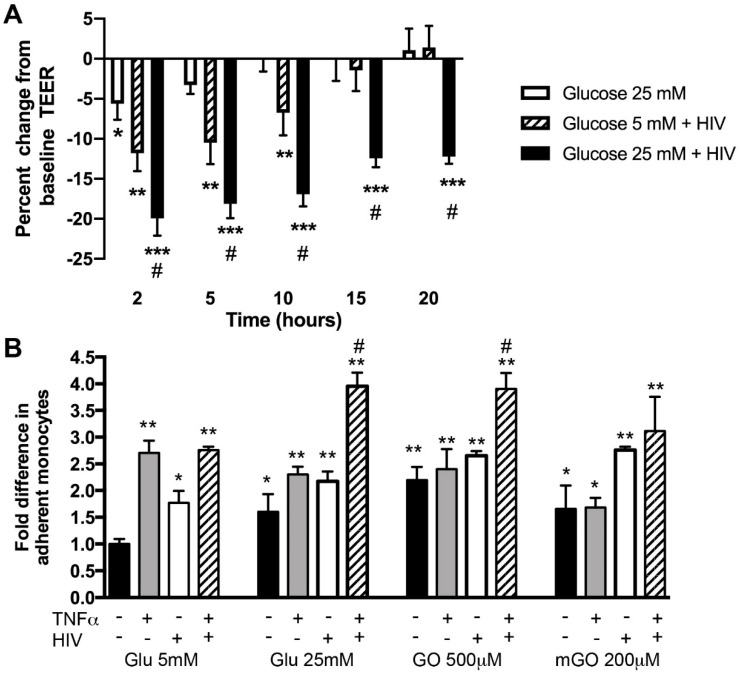
Hyperglycemia and HIV treatment diminish BBB tightness (**A**) and intensify primary human monocyte adhesion (**B**) to BMVEC (primary human brain endothelial cells). BMVEC were maintained in medium containing 5 mM glucose until a confluent monolayer was achieved (400–600 Ohms). BMVEC were exposed to glucose for 1 hr followed by HIV treatment at low or high dose, 17 and 35 ng/mL HIV p24, respectively, mimicking viremia [20,35]. TEER (transendothelial electrical resistance) was measured for 72 hr. Results are presented as percent change from baseline TEER, where 100% is defined as TEER values of non-treated (NT) cultures. Experiments were repeated twice with BMVEC from different donors and means +/− SEM of triplicate determinations are shown. For adhesion assay, BMVEC were treated for 1 hr with glucose, mGO (methylglyoxal) or GO (glyoxal), followed by overnight treatment with HIV (low dose) in the presence or absence of TNFα (75 ng/mL). Treatments were removed prior to addition of monocytes. Experiments were repeated twice with BMVEC and monocytes from different donors. Data are shown as fold difference (means ± SEM) of adhesion from triplicate determinations, with adhesion to untreated BMVEC assigned a value of 1. * *p* < 0.05, ** *p* < 0.01, *** *p* < 0.001 shows significant change vs. non-treated cells, whereas # *p* < 0.05 shows significant change vs. HIV and TNFα-stimulated cells in normal glucose (5 mM).

To mimic BBB injury in HIV-infected patients with DM conditions in vitro, we modeled it in the in vitro BBB model previously established in our laboratory with primary human brain endothelial cells (BMVEC) [30,31,32,33,34] using a combination of high glucose (HG) and infectious HIV-1_ADA_ (in two concentrations, of 17 and 35 ng/mL HIV p24 mimicking viremia) [20,35], and measured endothelial function utilizing TEER (transendothelial electrical resistance) technology. Exposure of BMVEC to HIV resulted in an immediate drop of TEER and, while there was recovery with lower HIV concentration, 17 ng/mL p24 (95%), the higher amount (35 ng/mL p24) of HIV led to continuous barrier disruption (82–85% of control) (Supplemental Figure S2). HG conditions showed a further decline in endothelial barrier tightness (up to 70% of control, Figure 2A). Next, we investigated whether DM conditions (HG and AGEs) [3], TNFα (increased in blood of infected patients [36]) and HIV_ADA_ would enhance adhesion of primary human monocytes to BMVEC monolayers. HIV_ADA_ was isolated from PBMC of a patient with AIDS, and has been previously described to play a role in BBB dysfunction [20,37,38]. Treatment with HIV_ADA_ or TNFα increases adhesion 2–2.5-fold. HG also increased adhesion (1.8-fold) and combination with HIV or TNFα further augmented adhesion (up to 2.2-fold) (Figure 2B). Combination of all three stimuli further enhanced adhesion to 3.9-fold, indicating synergistic effects. Similar results were observed with two AGEs (showing amplified adhesion by 3–3.5-fold) (Figure 2B).

**Figure 3:**
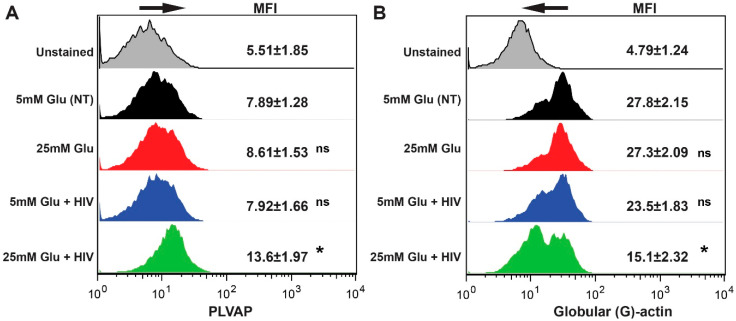
HIV and hyperglycemia induce PLVAP (plasmalemma vesicle associated protein) expression and actin rearrangement in BMVEC. Cells were stimulated with 25 mM Glu, 200 μM mGO or 1 mM GO alone or in combination with HIV (17 ng/mL HIV p24) [20,35]. Cells were labeled with fluorophore-labeled Abs and expression was measured by FACS. Data were collected from at least 20,000 events and repeated twice with BMVEC from different donors. Representative histograms for PLVAP (**A**) and G-actin (**B**) staining are shown, and mean fluorescent intensity (MFI) from three independent experiments are presented as the mean ± SEM. * *p* < 0.05 represent significance vs. non-stimulated cells.

Plasmalemma vesicle-associated protein (PLVAP) has been described as a marker of brain endothelial cell permeability [24,25,26]. We evaluated PLVAP expression in DM conditions alone and in conjunction with HIV_ADA_ exposure. There was a 2-fold increase of PLVAP in BMVEC exposed to HG and HIV_ADA_ (Figure 3A). Furthermore, we evaluated changes in the cytoskeleton in BMVEC by measuring globular actin (G-actin) after treatment with HG or HIV_ADA_ [39]. Treatment with HIV only resulted in slight decrease in G-actin-positive BMVEC (small hump in the left part of the respective histogram shows two populations of cells), whereas only the combination of HG and HIV led to a significant decrease of G-actin (higher hump in the left part of the respective histogram shows two populations of cells), indicating changes in cytoskeleton associated with diminished barrier tightness [39] (Figure 3B). Taken together, these results indicate a potential mechanism for barrier instability.

### 2.3. Hyperglycemia and HIV Exposure Reduce PDGF-Rβ Expression and Stimulate Actin Cytoskeletal Rearrangements in Primary Human Pericytes

**Figure 4:**
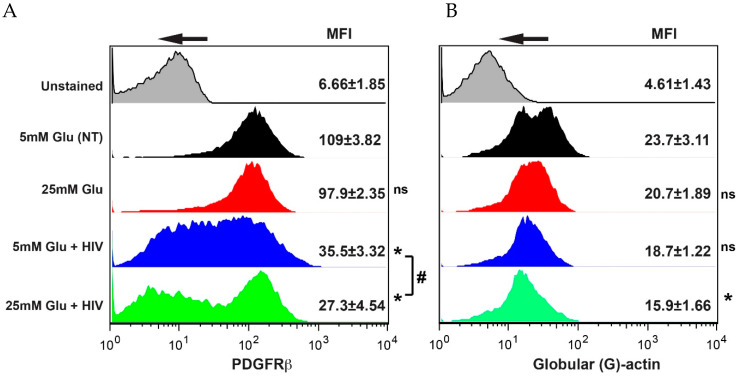
HIV and hyperglycemia diminish PDGF-Rβ expression and induce actin rearrangement in pericytes. Cells were stimulated with 25 mM Glu alone or in combination with HIV (17 ng/mL HIV p24) [20,35]. Cells were labeled with fluorophore-labeled Abs and expression was measured by FACS. MFI from three independent experiments are shown as the mean ± SEM. Data were collected from at least 20,000 events, and each repeat was performed with pericytes from a different donor. * *p* < 0.05 represent significance vs. non-stimulated cells, # *p* < 0.05 significance compared HIV-treated cells in normal vs. hyperglycemic conditions.

Expression of the key pericyte receptor PDGF-Rβ assures proper pericyte function and BBB integrity [3,4,27]. We assessed PDGF-Rβ expression after treatment with HIV and/or HG. While HG alone did not have an effect, HIV significantly downregulated PDGF-Rβ expression (depicted by a stretched histogram pick, representing cells with different levels of protein expression) and combination with HG further significantly diminished its expression (portrayed by two separate histogram picks, representing two populations of cells, one with initial expression level and the other close to unstained) (Figure 4A). Total MFIs (mean fluorescent intensities) of the treatment with HIV only or combination with HG were shown to be significant when compared to the normal glucose conditions or HG only. We detected the effects of HG and HIV on cytoskeleton in pericytes and showed that only the combination of HG and HIV diminished G-actin content in pericytes, which was associated with a less stable barrier (Figure 4B).

### 2.4. Exposure to HIV and Hyperglycemia Stimulates Expression of Adhesion Molecules on Both Primary Endothelial Cells and Pericytes

**Figure 5:**
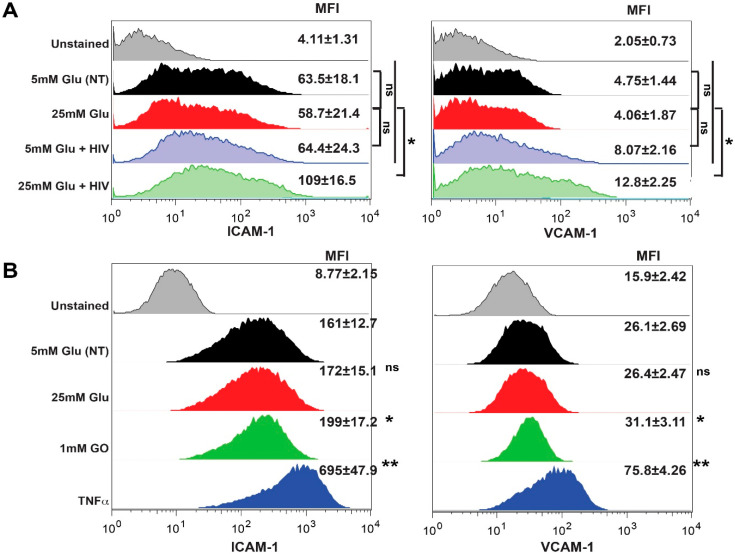
HIV and hyperglycemia induce ICAM-1 and VCAM-1 expression in BMVEC (**A**) and pericytes (**B**). Cells were stimulated with 25 mM Glu alone or in combination with HIV (17 ng/mL HIV p24) [20,35]. Cells were labeled with fluorophore-labeled Abs and expression was measured by FACS. Data were collected from at least 20,000 events and repeated twice with BMVEC or PC each time from a different donor. MFI from three independent experiments are shown as the mean ± SEM. * *p* < 0.05, ** *p* < 0.01 represent significance vs. non-stimulated cells.

Since we saw increased adhesion of primary monocytes to brain endothelial monolayer in Figure 2B, we decided to assess whether HG and HIV would increase the expression of adhesion molecules in BMVEC (Figure 5A). The adhesion molecules, intercellular adhesion molecule 1 (ICAM-1) and vascular cell adhesion molecule 1 (VCAM-1), both members of the Ig superfamily, have been demonstrated to be upregulated on the endothelial cells or pericytes of cerebral vessels [20,32,34,40,41,42,43,44]. Both ICAM-1 and VCAM-1 were demonstrated to mediate the adhesion of inflammatory cells via their respective ligands LFA-1 (αLβ2-integrin) and the α4-integrins, α4β1 and α4β7 to the inflamed cerebral vessels in vitro [40,45,46]. Indeed, the combination of HG and HIV upregulated ICAM-1 and VCAM-1 expression (*p* > 0.05). As pericyte expression of adhesion molecules may play a role in propagation of neuroinflammation, we treated pericytes in a similar fashion and demonstrated significant enhancement in ICAM-1 and VCAM-1 expression with combined HG and HIV exposure (Figure 5B).

## 3. Discussion

Despite introduction of ART keeping virus replication under control, it was noticed that a portion of HIV-infected people show a decline in brain function and movement skills, as well as shifts in behavior and mood. In addition to its effect on the immune system, HIV also affects the nervous system and the brain, which can diminish cognitive reserve in a number of ways. First, HIV can cross the BBB and infect glial cells, causing their dysfunction. Glial cells are needed to support neuronal health and their failure results in neuronal dysfunction. Second, HIV infection is also considered an inflammatory/neuroinflammatory disease associated with neurotoxin production. In general, such neuroinflammation has been shown in several clinical populations to reduce cognitive reserve and induce cognitive impairments [47]. A convergence of different factors such as a dietary change, inactive lifestyle and an aging population in Western countries has led to a fast escalation in the incidence of DM type 2 that carries a burden in terms of health and economic outcomes. Increasingly, type 2 diabetes is recognized as a major contributor to cognitive decline and dementia in older adults [48,49,50]. The more complex and sophisticated the connections between neurons are, the better they are able to communicate and support cognition, even in lieu of disease-related insults. Consequently, more studies are required to tackle the mechanisms affecting cognitive function in diseases such as DM alone or in conjunction with HIV infection. Dysfunction of the BBB has been associated with the development of dementia, specifically in DM patients [8,9,10,14], and HIV [5,17,18,36,51,52,53]. A recent study showed a correlation to the BBB disruption in HAND [54].

In the current study, we tested whether the presence of both conditions would lead to additive effects on BBB function. Our results show that the combination of both hyperglycemia or high levels of advanced glycation end products (AGEs) and HIV resulted in decreased tightness of the barrier. DM conditions in conjunction with HIV_ADA_ exposure showed a significant increase in PLVAP expression. PLVAP has been portrayed as a marker of brain endothelial cell permeability [24,25,26]. Interestingly, DM conditions or HIV exposure alone did not produce any effect on PLVAP expression. Similar effects were observed on actin cytoskeletal rearrangements. There were actin cytoskeletal rearrangements in pericytes paralleling the combined actions of HG and HIV in brain endothelial cells, indicating that changes in the cytoskeleton were associated with diminished BBB tightness [39].

Since the expression of the key pericyte receptor PDGF-Rβ assures proper pericyte function and barrier integrity [3,27], we tested its expression after treatment with HIV and/or HG. Although HG alone did not have an effect, HIV exposure significantly downregulated PDGF-Rβ expression and combination with HG further significantly diminished its expression. Cell adhesion molecules (CAMs) play an important role in leukocyte adhesion and subsequently increase myeloid cell extravasation, causing further vascular leak. The current study shows that the combination of HG and HIV upregulated ICAM-1 and VCAM-1 in both endothelial cells and pericytes in vitro and in the most prominent reduction of claudin-5 expression and decreased pericyte coverage in vivo. Decreased TJ protein expression has been shown to be associated with BBB injury within HIV-driven chronic inflammation [28,29] and DM conditions [9,55,56,57,58]. Disruption of the BBB and its association with cognitive decline has been also exhibited in DM patients [14] and DM mouse models [3,4]. It was accompanied by diminished TJ protein expression in brain endothelium and attenuated staining for pericyte marker [3,4]. Changes in Toll-like receptors (TLRs), that serve as key innate immune receptors, and inflammatory mediators such as TNFα, IL-6, MCP-1 and IL-1β, have been linked with DM [59,60]. Cytokines, such as TNFα, IL-6, IL-1β, IL-4, IL-10 and TGF-β, were shown to be highly elevated in the serum of HIV patients prior to ART treatment, while TNFα and TGF-β remained significantly increased even 12 months after ART therapy [61]. Most of the cytokines mentioned above were shown to lead to BBB dysfunction by themselves [62,63], but their presence also results in endothelial activation and production of adhesion molecules, such as VCAM-1 and ICAM-1 [43,63,64,65,66], allowing an increase in leukocyte adhesion and subsequent migration across the BBB, leading to further BBB dysfunction. Since barrier compromise exists in DM and HIV infection, and it is aggravated by a combination of both, there is a possibility of the same cellular and molecular targets.

Endothelial dysfunction initiated by HIV infection has been acknowledged in the literature as a critical connection between infection, immune activation, inflammation and different cardiovascular complications. Since HIV cannot actively replicate in endothelial cells, endothelial dysfunction is contingent on both inflammatory mediators released in the microenvironment by HIV-infected cells as well as on HIV-encoded proteins. The HIV proteins, gp120 (envelope glycoprotein) and Tat (transactivator of transcription), are constantly secreted into the endothelial cell microenvironment during HIV infection [29,67,68,69]. However, another HIV-encoded protein, Nef, was shown to be transferred onto endothelial cells during HIV infection [67,70]. These proteins can have significant direct effects on the endothelium, such as increased leukocyte-adhesiveness, permeability, oxidative stress, apoptosis and stimulation of cytokine secretion [28,67,71,72,73,74,75,76]. All these effects contribute consequently to endothelial dysfunction. HIV-encoded proteins have been found to have harmful effects on pericytes too. We and others have demonstrated that pericyte exposure to HIV or cytokines (TNFα or IL-1β) is followed by declined secretion of factors such as angiopoietin-1 and TGF-β, which are known to promote BBB formation [20,77]. Buch and colleagues showed that HIV-Tat-mediated migration of pericytes was dependent on PDGF-Rβ signaling and ultimately led to loss of pericyte coverage from the endothelium, with a subsequent breach of the BBB [77]. A decrease in pericyte coverage has been reported in AD, MS, ALS, diabetic microangiopathy, and stroke [78,79,80,81], while very little is known about pericyte changes in the brain tissue of HIV-1-infected patients. Our previous data indicate reduced coverage and/or downregulation of pericyte markers in patients with HIV infection, untreated or on ART, even without HIV-1 encephalitis [20,77].

Causes for vascular dysfunction in DM are related to heightened generation of ROS due to enhanced glucose metabolism, subsequent buildup of glycolytic intermediates, advanced glycation end products (AGEs) and activation of protein kinase C (PKC) [82,83]. These events eventually may result in NF-κB activation, overexpression of adhesion molecules (VCAM-1, ICAM-1), activation of the small GTPase, RhoA and diminished expression of TJ proteins [84]. Interestingly, our studies showed PKC and RhoA activation in human BMVEC play a significant role in BBB injury associated with HIV-driven chronic inflammation leading to decreases in TJ protein expression [28,29]. There are similarities in the appearance of injury, the same signaling pathways are involved, and there is strong evidence that the combination of both could lead to cognitive deficits (summarized in Graphical Abstract). However, very little information is currently available on the extent and mechanisms of BBB demise in the combination of HIV/DM and whether experimentally proven strategies that protect the barrier can prevent cognitive decline. In this study, we identified functional effects leading to BBB disruption in both conditions and more studies are needed to identify therapeutic compounds to challenge these problems.

## 4. Materials and Methods

### 4.1. Cells

Primary BMVEC, isolated from vessels from brain resection tissue (showing no abnormalities) from patients undergoing surgery for treatment of intractable epilepsy, were supplied by Michael Bernas and Dr. Marlys Witte (University of Arizona, Tucson, AZ, USA) and maintained as described [32,44]. Primary human brain pericytes were isolated by us and cultured as described [20].

### 4.2. Determination of HIV-1 p24 Concentration

Concentration of p24 antigen was measured in HIV-1-containing supernatant with the HIV-1 p24 Antigen Capture Assay (Advanced BioScience Laboratories, Inc., Kensington, MD, USA), according to manufacturer’s instructions. Assuming that an HIV core is composed of 2000 p24 capsid molecules, 1 ng of p24 antigen corresponds to 1.25 × 10^7^ viral particles [20,85].

### 4.3. Monocyte Adhesion Assays

Adhesion assays were performed as described [31,32,44]. BMVEC were pretreated for 1 hr with DM relevant stimuli, high glucose, glyoxal (GO) or methylglyoxal (mGO), followed by overnight treatment with HIV (low dose, (17 ng/mL HIV p24) [20,35]) and stimulation with TNFα (75 ng/mL) for 1 hr. Treatments were removed prior to monocyte introduction. Fluorescently-labeled monocytes (2.5 × 10^5^ cells/well) were added to the endothelial monolayers for 15 min at 37 °C. After adhesion, monolayers were washed and relative fluorescence of the attached monocytes was acquired on a fluorescence plate reader, Synergy 2 plate reader (BioTek, Winooski, VT, USA). Results are presented as the mean ± SEM fold adhesion (number of adherent monocytes for each experimental condition divided by the basal adhesion of the untreated control). 

### 4.4. Transendothelial Electrical Resistance (TEER) 

BMVEC were plated on collagen type I coated 96W20idf electrode arrays (Applied Biophysics, Troy, NY, USA) and were treated for 1 hr with glucose, followed by overnight treatment with HIV (low or high dose, 17 and 35 ng/mL HIV p24, respectively, mimicking viremia) for 24 hr [20,35]. TEER measurements were performed using the 1600R ECIS System (Applied Biophysics, Troy, NY, USA) as described [31,32,44]. The results are presented as the average percent change from baseline TEER (expressed as average ± SEM) from at least three independent experiments consisting of four to six replicates each.

### 4.5. ICAM-1, VCAM-1, PLVAP, PDGF-Rβ Expression and G-actin Quantification by Flow Cytometry

Analysis of surface expression of adhesion molecules was performed using the conjugated antibodies ICAM-1-APC and vascular cell adhesion molecule 1 (VCAM-1)-FITC (BD Biosciences, San Jose, CA, USA). Plasmalemma vesicle associated protein (PLVAP) and PDGF-Rβ proteins were stained with anti-PLVAP-FITC and anti-PDGF-Rβ -APC (Novus Biologicals, LLC., Centennial, CO, USA). For G-actin quantification, cells after stimulation were washed with ice-cold PBS and fixed for 10 min as described [35]. Globular actin (G-actin) was stained with DNase 1-Alexa 488 (Life Technologies), according to the manufacturer’s instructions. Following staining, data were acquired with a FACS BD Canto II flow cytometer (BD Biosciences) and analyzed with FlowJo software v9.9.6 (Tree Star, Inc., Ashland, OR, USA). Data were collected from at least 20,000 events and repeated twice with BMVEC or pericytes each time from different donors. The mean fluorescent intensity (MFI) of stain was calculated in a cell population and presented as average ± SEM. 

### 4.6. Human Brain Tissues

The current study included two patient cohorts for a total of 26 HIV-seropositive cases and 16 seronegative cases. The first cohort described consisted of a total of 21 HIV-seropositive cases and 5 HIV-seronegative patients chosen as a control group (matched for age, gender and racial status, autopsy cases from the Temple University Hospital). The second cohort was cases provided by NNTC and consisted of 8 HIV-seropositive cases with diabetes (6 ART-treated and two untreated) and 11 seronegative cases with diabetes. Available demographic (age, gender, race/ethnicity) and clinical information (ART status, CD4 count, co-morbidity and neurocognition) were collected from hospital medical records and information provided by NNTC (Table 1). This study has been approved by the Institutional Review Board of Temple University School of Medicine according to the ethical guidelines of the Helsinki Declaration of 1975 (and as revised in 1983). Macroscopic and microscopic examination of the brains used a standardized protocol with sections from the neocortex (frontal and parietal), basal ganglia, hippocampus, midbrain, pons, medulla, cerebellum, spinal cord and any grossly evident lesion. Paraffin sections (5 *μ*m) from frontal cortex and basal ganglia were stained with hematoxylin-eosin. One section from the frontal cortical lobe and the hippocampus from each case were used for the evaluation of neuroinflammation and BBB structure by immunohistochemistry using the following antibodies: anti-CD13 for pericytes (1:1000, R&D Systems, Minneapolis, MN, USA). Primary antibodies were detected by Vectastain Elite Kit (Vector Laboratories, Burlingame, CA, USA) with DAB or Vector Blue substrate. In addition, double immunostains were performed on frozen sections by indirect immunofluorescence for claudin-5, tight junction (TJ) protein, in brain endothelium (1:50, Zymed/LifeTechnologies, Grand Island, NY, USA) and PDGFR-β, pericyte marker (1:50, Santa Cruz Biotechnology Inc., Santa Cruz, CA, USA) as described [75]. Tissue sections were rinsed, and secondary antibodies conjugated to Alexa-488 or to rhodamine (diluted 1:250, Invitrogen) were then added for 1 h. Immunostains for CD13 and claudin-5 were assessed in blinded fashion using a semi-quantitative score protocol: 0—negative staining, 1—mild positivity (<10% of cells), 2—moderate positivity (10–50%), 3—strong positivity (50–75%) and 4—very strong (75–100%).

### 4.7. Statistical Analysis

Data are expressed as the mean ± SEM of experiments conducted multiple times. Statistical analyses were performed utilizing Prism v8.3.0 software (GraphPad Software Inc., San Diego, CA, USA). Comparisons between several groups were performed by two-way analysis of variance with Tukey post hoc tests (TEER and migration assays). Alternatively, one-way analysis of variance with Tukey post hoc tests (FACS and IHC). Differences were considered significant at *p* < 0.05.

## 5. Conclusions

In this manuscript, we uncovered a decrease in pericyte coverage and expression of tight junction proteins in human brain tissues from HIV patients with DM and evidence of HAND when compared to HIV-infected patients without DM or seronegative DM patients. Exploiting our in vitro BBB models, we confirmed diminution of barrier integrity, enhanced monocyte adhesion, changes in cytoskeleton and overexpression of adhesion molecules in primary human brain endothelial cells or human brain pericytes after exposure to HIV and DM-relevant stimuli. Our study presents for the first-time evidence of compromised BBB function in HIV-DM patients and indicates on potential mechanisms leading to it in brain endothelium and pericytes that may result in poorer cognitive performance compared to individuals without HIV and DM.

## Figures and Tables

**Table 1:** Patient demographic and clinical information.

	HIV with ART (11)	HIV without ART (10)	HIV with Diabetes (8, ART - 6, without ART - 2)	HIV Naive with Diabetes (11)	HIV Naïve (5)
**Age**					
<35	0	1	0	0	1
36–64	10	0	6	8	3
>64	1	1	2	3	1
**Gender**					
Male	5	7	6	6	3
Female	5	3	2	5	2
**Ethnicity**					
Caucasian	1	1	4	6	1
African					
American	6	6	2	3	2
Hispanic	4	3	2	2	2
**Comorbidity**					
Hepatitis C	3	2	0	0	1
Hepatitis B	1	0	0	0	0
Hepatitis B and C	0	1	0	0	0

## Supplementary Materials

The following are available online at https://www.mdpi.com/1422-0067/21/13/4663/s1.

## References

[1] Snyder E.L., Stramer S.L., Benjamin R.J. (2015). The Safety of the Blood Supply--Time to Raise the Bar. N. Engl. J. Med..

[2] Kisler K., Nelson A.R., Rege S.V., Ramanathan A., Wang Y., Ahuja A., Lazic D., Tsai P.S., Zhao Z., Zhou Y. (2017). Pericyte degeneration leads to neurovascular uncoupling and limits oxygen supply to brain. Nat. Neurosci..

[3] Rom S., Heldt N.A., Gajghate S., Seliga A., Reichenbach N., Persidsky Y. (2020). Hyperglycemia and advanced glycation end products disrupt BBB and promote occludin and claudin-5 protein secretion on extracellular microvesicles. Sci. Rep..

[4] Rom S., Zuluaga-Ramirez V., Gajghate S., Seliga A., Winfield M., Heldt N.A., Kolpakov M.A., Bashkirova Y.V., Sabri A.K., Persidsky Y. (2019). Hyperglycemia-Driven Neuroinflammation Compromises BBB Leading to Memory Loss in Both Diabetes Mellitus (DM) Type 1 and Type 2 Mouse Models. Mol. Neurobiol..

[5] Ellis R.J., Badiee J., Vaida F., Letendre S., Heaton R.K., Clifford D., Collier A.C., Gelman B., McArthur J., Morgello S. (2011). CD4 nadir is a predictor of HIV neurocognitive impairment in the era of combination antiretroviral therapy. AIDS.

[6] Kraft-Terry S.D., Stothert A.R., Buch S., Gendelman H.E. (2010). HIV-1 neuroimmunity in the era of antiretroviral therapy. Neurobiol. Dis..

[7] Thompson K.A., Cherry C.L., Bell J.E., McLean C.A. (2011). Brain Cell Reservoirs of Latent Virus in Presymptomatic HIV-Infected Individuals. Am. J. Pathol..

[8] Moran C., Tapp R.J., Hughes A.D., Magnussen C.G., Blizzard L., Phan T.G., Beare R., Witt N., Venn A., Munch G. (2016). The Association of Type 2 Diabetes Mellitus with Cerebral Gray Matter Volume Is Independent of Retinal Vascular Architecture and Retinopathy. J. Diabetes Res..

[9] Prasad S., Sajja R.K., Naik P., Cucullo L. (2014). Diabetes Mellitus and Blood-Brain Barrier Dysfunction: An Overview. J. Pharmacovigil.

[10] Sutherland G.T., Lim J., Srikanth V., Bruce D.G. (2017). Epidemiological Approaches to Understanding the Link Between Type 2 Diabetes and Dementia. J. Alzheimer’s Dis. JAD.

[11] Di Marco E., Jha J.C., Sharma A., Wilkinson-Berka J.L., Jandeleit-Dahm K.A., de Haan J.B. (2015). Are reactive oxygen species still the basis for diabetic complications?. Clin. Sci. (London, England: 1979).

[12] van Bussel F.C.G., Backes W.H., Hofman P.A.M., van Oostenbrugge R.J., van Boxtel M.P.J., Verhey F.R.J., Steinbusch H.W.M., Schram M.T., Stehouwer C.D.A., Wildberger J.E. (2017). Cerebral Pathology and Cognition in Diabetes: The Merits of Multiparametric Neuroimaging. Front. Neurosci..

[13] Imamine R., Kawamura T., Umemura T., Umegaki H., Kawano N., Hotta M., Kouchi Y., Hatsuda S., Watarai A., Kanai A. (2011). Does cerebral small vessel disease predict future decline of cognitive function in elderly people with type 2 diabetes?. Diabetes Res. Clin. Pract..

[14] Janelidze S., Hertze J., Nagga K., Nilsson K., Nilsson C., Wennstrom M., van Westen D., Blennow K., Zetterberg H., Hansson O. (2017). Increased blood-brain barrier permeability is associated with dementia and diabetes but not amyloid pathology or APOE genotype. Neurobiol. Aging.

[15] Sharma B., Singh N. (2010). Pitavastatin and 4’-hydroxy-3’-methoxyacetophenone (HMAP) reduce cognitive dysfunction in vascular dementia during experimental diabetes. Curr. Neurovascular Res..

[16] Stranahan A.M., Hao S., Dey A., Yu X., Baban B. (2016). Blood-brain barrier breakdown promotes macrophage infiltration and cognitive impairment in leptin receptor-deficient mice. J. Cereb. Blood Flow Metab..

[17] Sacktor N. (2018). Changing clinical phenotypes of HIV-associated neurocognitive disorders. J. Neurovirol.

[18] Valdez A.N., Rubin L.H., Neigh G.N. (2016). Untangling the Gordian knot of HIV, stress, and cognitive impairment. Neurobiol. Stress.

[19] Maki P.M., Rubin L.H., Valcour V., Martin E., Crystal H., Young M., Weber K.M., Manly J., Richardson J., Alden C. (2015). Cognitive function in women with HIV: Findings from the Women’s Interagency HIV Study. Neurology.

[20] Persidsky Y., Hill J., Zhang M., Dykstra H., Winfield M., Reichenbach N.L., Potula R., Mukherjee A., Ramirez S.H., Rom S. (2016). Dysfunction of brain pericytes in chronic neuroinflammation. J. Cereb. Blood Flow Metab..

[21] Vance D.E., Fazeli P.L., Dodson J.E., Ackerman M., Talley M., Appel S.J. (2014). The synergistic effects of HIV, diabetes, and aging on cognition: Implications for practice and research. J. Neurosci. Nurs..

[22] Saylor D., Dickens A.M., Sacktor N., Haughey N., Slusher B., Pletnikov M., Mankowski J.L., Brown A., Volsky D.J., McArthur J.C. (2016). HIV-associated neurocognitive disorder - pathogenesis and prospects for treatment. Nat. Rev. Neurol.

[23] NIMH NeuroHIV in the ART era. https://www.nimh.nih.gov/news/events/2017/neurohiv-in-the-art-era.shtml.

[24] Liebner S., Corada M., Bangsow T., Babbage J., Taddei A., Czupalla C.J., Reis M., Felici A., Wolburg H., Fruttiger M. (2008). Wnt/beta-catenin signaling controls development of the blood-brain barrier. J. Cell Biol..

[25] Bosma E.K., van Noorden C.J.F., Schlingemann R.O., Klaassen I. (2018). The role of plasmalemma vesicle-associated protein in pathological breakdown of blood-brain and blood-retinal barriers: Potential novel therapeutic target for cerebral edema and diabetic macular edema. Fluids Barriers CNS.

[26] Guo L., Zhang H., Hou Y., Wei T., Liu J. (2016). Plasmalemma vesicle-associated protein: A crucial component of vascular homeostasis. Exp. Ther. Med..

[27] Hill J., Rom S., Ramirez S.H., Persidsky Y. (2014). Emerging Roles of Pericytes in the Regulation of the Neurovascular Unit in Health and Disease. J. Neuroimmune Pharmacol..

[28] Persidsky Y., Heilman D., Haorah J., Zelivyanskaya M., Persidsky R., Weber G.A., Shimokawa H., Kaibuchi K., Ikezu T. (2006). Rho-mediated regulation of tight junctions during monocyte migration across the blood-brain barrier in HIV-1 encephalitis (HIVE). Blood.

[29] Kanmogne G.D., Schall K., Leibhart J., Knipe B., Gendelman H.E., Persidsky Y. (2007). HIV-1 gp120 compromises blood-brain barrier integrity and enhance monocyte migration across blood-brain barrier: Implication for viral neuropathogenesis. J. Cereb. Blood Flow Metab..

[30] Ramirez S.H., Fan S., Dykstra H., Rom S., Mercer A., Reichenbach N.L., Gofman L., Persidsky Y. (2013). Inhibition of Glycogen Synthase Kinase 3beta Promotes Tight Junction Stability in Brain Endothelial Cells by Half-Life Extension of Occludin and Claudin-5. PLoS ONE.

[31] Rom S., Dykstra H., Zuluaga-Ramirez V., Reichenbach N.L., Persidsky Y. (2015). miR-98 and let-7g* protect the blood-brain barrier under neuroinflammatory conditions. J. Cereb. Blood Flow Metab..

[32] Rom S., Fan S., Reichenbach N., Dykstra H., Ramirez S.H., Persidsky Y. (2012). Glycogen synthase kinase 3beta inhibition prevents monocyte migration across brain endothelial cells via Rac1-GTPase suppression and down-regulation of active integrin conformation. Am. J. Pathol..

[33] Rom S., Persidsky Y. (2013). Cannabinoid receptor 2: Potential role in immunomodulation and neuroinflammation. J. Neuroimmune Pharmacol..

[34] Rom S., Zuluaga-Ramirez V., Dykstra H., Reichenbach N., Ramirez S.H., Persidsky Y. (2015). Poly(ADP-ribose) polymerase-1 inhibition in brain endothelium protects the blood–brain barrier under physiologic and neuroinflammatory conditions. J. Cereb. Blood Flow Metab..

[35] Rom S., Reichenbach N.L., Dykstra H., Persidsky Y. (2015). The dual action of poly(ADP-ribose) polymerase -1 (PARP-1) inhibition in HIV-1 infection: HIV-1 LTR inhibition and diminution in Rho GTPase activity. Front. Microbiol..

[36] Farhadian S., Patel P., Spudich S. (2017). Neurological Complications of HIV Infection. Curr. Infect. Dis. Rep..

[37] Ghorpade A., Nukuna A., Che M., Haggerty S., Persidsky Y., Carter E., Carhart L., Shafer L., Gendelman H.E. (1998). Human immunodeficiency virus neurotropism: An analysis of viral replication and cytopathicity for divergent strains in monocytes and microglia. J. Virol..

[38] Gendelman H.E., Orenstein J.M., Martin M.A., Ferrua C., Mitra R., Phipps T., Wahl L.A., Lane H.C., Fauci A.S., Burke D.S. (1988). Efficient isolation and propagation of human immunodeficiency virus on recombinant colony-stimulating factor 1-treated monocytes. J. Exp. Med..

[39] Rom S., Zuluaga-Ramirez V., Reichenbach N.L., Dykstra H., Gajghate S., Pacher P., Persidsky Y. (2016). PARP inhibition in leukocytes diminishes inflammation via effects on integrins/cytoskeleton and protects the blood-brain barrier. J. Neuroinflammation.

[40] Engelhardt B., Wolburg-Buchholz K., Wolburg H. (2001). Involvement of the choroid plexus in central nervous system inflammation. Microsc. Res. Tech..

[41] Dietrich J.B. (2002). The adhesion molecule ICAM-1 and its regulation in relation with the blood-brain barrier. J. Neuroimmunol..

[42] Sans E., Delachanal E., Duperray A. (2001). Analysis of the roles of ICAM-1 in neutrophil transmigration using a reconstituted mammalian cell expression model: Implication of ICAM-1 cytoplasmic domain and Rho-dependent signaling pathway. J. Immunol..

[43] Yang L., Froio R.M., Sciuto T.E., Dvorak A.M., Alon R., Luscinskas F.W. (2005). ICAM-1 regulates neutrophil adhesion and transcellular migration of TNF-alpha-activated vascular endothelium under flow. Blood.

[44] Rom S., Zuluaga-Ramirez V., Dykstra H., Reichenbach N.L., Pacher P., Persidsky Y. (2013). Selective activation of cannabinoid receptor 2 in leukocytes suppresses their engagement of the brain endothelium and protects the blood-brain barrier. Am. J. Pathol..

[45] Hyun Y.M., Chung H.L., McGrath J.L., Waugh R.E., Kim M. (2009). Activated integrin VLA-4 localizes to the lamellipodia and mediates T cell migration on VCAM-1. J. Immunol..

[46] Lim Y.C., Wakelin M.W., Henault L., Goetz D.J., Yednock T., Cabanas C., Sanchez-Madrid F., Lichtman A.H., Luscinskas F.W. (2000). Alpha4beta1-integrin activation is necessary for high-efficiency T-cell subset interactions with VCAM-1 under flow. Microcirculation.

[47] Kamat A., Misra V., Cassol E., Ancuta P., Yan Z., Li C., Morgello S., Gabuzda D. (2012). A plasma biomarker signature of immune activation in HIV patients on antiretroviral therapy. PLoS ONE.

[48] Fried P.J., Pascual-Leone A., Bolo N.R. (2019). Diabetes and the link between neuroplasticity and glutamate in the aging human motor cortex. Clin. Neurophysiol..

[49] Cui Y., Liang X., Gu H., Hu Y., Zhao Z., Yang X.Y., Qian C., Yang Y., Teng G.J. (2017). Cerebral perfusion alterations in type 2 diabetes and its relation to insulin resistance and cognitive dysfunction. Brain Imaging Behav..

[50] Umegaki H. (2014). Type 2 diabetes as a risk factor for cognitive impairment: Current insights. Clin. Interv. Aging.

[51] Boven L.A., Gomes L., Hery C., Gray F., Verhoef J., Portegies P., Tardieu M., Nottet H.S. (1999). Increased peroxynitrite activity in AIDS dementia complex: Implications for the neuropathogenesis of HIV-1 infection. J. Immunol..

[52] Persidsky Y., Zheng J., Miller D., Gendelman H.E. (2000). Mononuclear phagocytes mediate blood-brain barrier compromise and neuronal injury during HIV-1-associated dementia. J. Leukoc. Biol..

[53] Persidsky Y., Limoges J., Rasmussen J., Zheng J., Gearing A., Gendelman H.E. (2001). Reduction in glial immunity and neuropathology by a PAF antagonist and an MMP and TNFalpha inhibitor in SCID mice with HIV-1 encephalitis. J. Neuroimmunol..

[54] Chaganti J., Marripudi K., Staub L.P., Rae C.D., Gates T.M., Moffat K.J., Brew B.J. (2019). Imaging correlates of the blood-brain barrier disruption in HIV-associated neurocognitive disorder and therapeutic implications. AIDS.

[55] Chehade J.M., Haas M.J., Mooradian A.D. (2002). Diabetes-related changes in rat cerebral occludin and zonula occludens-1 (ZO-1) expression. Neurochem. Res..

[56] Huber J.D., VanGilder R.L., Houser K.A. (2006). Streptozotocin-induced diabetes progressively increases blood-brain barrier permeability in specific brain regions in rats. Am. J. Physiol. Heart Circ. Physiol.

[57] Hawkins B.T., Lundeen T.F., Norwood K.M., Brooks H.L., Egleton R.D. (2007). Increased blood-brain barrier permeability and altered tight junctions in experimental diabetes in the rat: Contribution of hyperglycaemia and matrix metalloproteinases. Diabetologia.

[58] Mogi M., Horiuchi M. (2011). Neurovascular coupling in cognitive impairment associated with diabetes mellitus. Circ. J. Off. J. Jpn. Circ. Soc..

[59] Gupta S., Maratha A., Siednienko J., Natarajan A., Gajanayake T., Hoashi S., Miggin S. (2017). Analysis of inflammatory cytokine and TLR expression levels in Type 2 Diabetes with complications. Sci. Rep..

[60] Xiao J., Li J., Cai L., Chakrabarti S., Li X. (2014). Cytokines and diabetes research. J. Diabetes Res..

[61] Osuji F.N., Onyenekwe C.C., Ahaneku J.E., Ukibe N.R. (2018). The effects of highly active antiretroviral therapy on the serum levels of pro-inflammatory and anti-inflammatory cytokines in HIV infected subjects. J. Biomed. Sci..

[62] Stamatovic S.M., Keep R.F., Kunkel S.L., Andjelkovic A.V. (2003). Potential role of MCP-1 in endothelial cell tight junction ‘opening’: Signaling via Rho and Rho kinase. J. Cell Sci..

[63] Ozaki H., Ishii K., Horiuchi H., Arai H., Kawamoto T., Okawa K., Iwamatsu A., Kita T. (1999). Cutting edge: Combined treatment of TNF-alpha and IFN-gamma causes redistribution of junctional adhesion molecule in human endothelial cells. J. Immunol..

[64] Zhang F., Yu W., Hargrove J.L., Greenspan P., Dean R.G., Taylor E.W., Hartle D.K. (2002). Inhibition of TNF-alpha induced ICAM-1, VCAM-1 and E-selectin expression by selenium. Atherosclerosis.

[65] Quagliaro L., Piconi L., Assaloni R., Da Ros R., Maier A., Zuodar G., Ceriello A. (2005). Intermittent high glucose enhances ICAM-1, VCAM-1 and E-selectin expression in human umbilical vein endothelial cells in culture: The distinct role of protein kinase C and mitochondrial superoxide production. Atherosclerosis.

[66] Van Wetering S., Van Den Berk N., Van Buul J.D., Mul F.P., Lommerse I., Mous R., Ten Klooster J.P., Zwaginga J.J., Hordijk P.L. (2003). VCAM-1-mediated Rac signaling controls endothelial cell-cell contacts and leukocyte transmigration. Am. J. Physiol. Cell Physiol..

[67] Anand A.R., Rachel G., Parthasarathy D. (2018). HIV Proteins and Endothelial Dysfunction: Implications in Cardiovascular Disease. Front. Cardiovasc. Med..

[68] D’Aversa T.G., Eugenin E.A., Berman J.W. (2005). NeuroAIDS: Contributions of the human immunodeficiency virus-1 proteins Tat and gp120 as well as CD40 to microglial activation. J. Neurosci. Res..

[69] Rom S., Pacifici M., Passiatore G., Aprea S., Waligorska A., Del Valle L., Peruzzi F. (2011). HIV-1 Tat binds to SH3 domains: Cellular and viral outcome of Tat/Grb2 interaction. Biochim. Biophys. Acta.

[70] van Marle G., Henry S., Todoruk T., Sullivan A., Silva C., Rourke S.B., Holden J., McArthur J.C., Gill M.J., Power C. (2004). Human immunodeficiency virus type 1 Nef protein mediates neural cell death: A neurotoxic role for IP-10. Virology.

[71] Chaudhuri A., Duan F., Morsey B., Persidsky Y., Kanmogne G.D. (2008). HIV-1 activates proinflammatory and interferon-inducible genes in human brain microvascular endothelial cells: Putative mechanisms of blood-brain barrier dysfunction. J. Cereb. Blood Flow Metab..

[72] Buckner C.M., Luers A.J., Calderon T.M., Eugenin E.A., Berman J.W. (2006). Neuroimmunity and the blood-brain barrier: Molecular regulation of leukocyte transmigration and viral entry into the nervous system with a focus on neuroAIDS. J. Neuroimmune Pharmacol..

[73] Avison M.J., Nath A., Greene-Avison R., Schmitt F.A., Greenberg R.N., Berger J.R. (2004). Neuroimaging correlates of HIV-associated BBB compromise. J. Neuroimmunol..

[74] Berger J.R., Avison M. (2004). The blood brain barrier in HIV infection. Front. Biosci.

[75] Ramirez S.H., Fan S., Dykstra H., Reichenbach N., Del Valle L., Potula R., Phipps R.P., Maggirwar S.B., Persidsky Y. (2010). Dyad of CD40/CD40 ligand fosters neuroinflammation at the blood-brain barrier and is regulated via JNK signaling: Implications for HIV-1 encephalitis. J. Neurosci..

[76] Zhong Y., Zhang B., Eum S.Y., Toborek M. (2012). HIV-1 Tat triggers nuclear localization of ZO-1 via Rho signaling and cAMP response element-binding protein activation. J. Neurosci..

[77] Niu F., Yao H., Zhang W., Sutliff R.L., Buch S. (2014). Tat 101-mediated enhancement of brain pericyte migration involves platelet-derived growth factor subunit B homodimer: Implications for human immunodeficiency virus-associated neurocognitive disorders. J. Neurosci..

[78] Sengillo J.D., Winkler E.A., Walker C.T., Sullivan J.S., Johnson M., Zlokovic B.V. (2012). Deficiency in mural vascular cells coincides with blood-brain barrier disruption in Alzheimer’s disease. Brain Pathol..

[79] Winkler E.A., Sengillo J.D., Sullivan J.S., Henkel J.S., Appel S.H., Zlokovic B.V. (2013). Blood-spinal cord barrier breakdown and pericyte reductions in amyotrophic lateral sclerosis. Acta Neuropathol..

[80] Murakami M. (2012). Signaling required for blood vessel maintenance: Molecular basis and pathological manifestations. Int. J. Vasc. Med..

[81] Vates G.E., Takano T., Zlokovic B., Nedergaard M. (2010). Pericyte constriction after stroke: The jury is still out. Nat. Med..

[82] Brownlee M. (2001). Biochemistry and molecular cell biology of diabetic complications. Nature.

[83] Dias I.H., Griffiths H.R. (2014). Oxidative stress in diabetes - circulating advanced glycation end products, lipid oxidation and vascular disease. Ann. Clin. Biochem..

[84] Lu Q.Y., Chen W., Lu L., Zheng Z., Xu X. (2014). Involvement of RhoA/ROCK1 signaling pathway in hyperglycemia-induced microvascular endothelial dysfunction in diabetic retinopathy. Int. J. Clin. Exp. Pathol..

[85] Vermeire J., Naessens E., Vanderstraeten H., Landi A., Iannucci V., Van Nuffel A., Taghon T., Pizzato M., Verhasselt B. (2012). Quantification of reverse transcriptase activity by real-time PCR as a fast and accurate method for titration of HIV, lenti- and retroviral vectors. PLoS ONE.

