# Supplementary material for: Combination of HIV-1 and Diabetes Enhances Blood Brain Barrier Injury via Effects on Brain Endothelium and Pericytes
Source: Int J Mol Sci. 2020 Jun 30;21(13):4663. doi: 10.3390/ijms21134663 (PMC7370277; doi:10.3390/ijms21134663)
Supplement: Supplementary file 1 [file ijms-21-04663-s001.pdf]

International Journal of *Molecular Sciences*

Supplemental material for the article "Combination of HIV-1 and diabetes enhances blood brain barrier injury via effects on brain endothelium and pericytes"

Slava Rom, Sachin Gajghate, Malika Winfield, Nancy L. Reichenbach and Yuri Persidsky

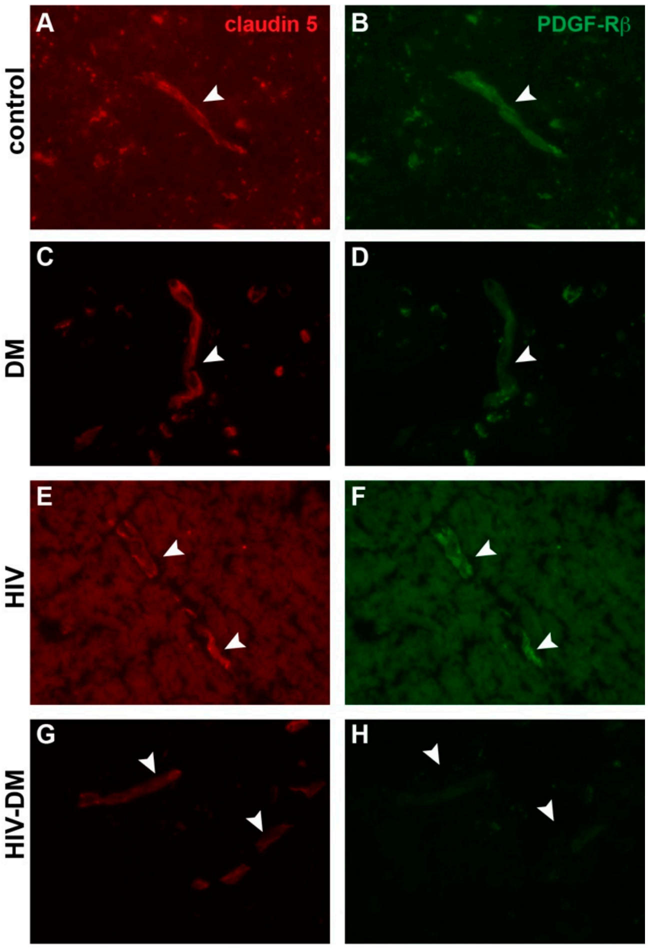

Supplemental Figure 1. *Decrease of pericyte coverage and claudin-5 expression at the BBB in DM, HIV-infected and HIV-infected patients with DM.* (A,B) Membrane-associated staining for claudin-5, TJ protein in brain endothelium (A), and strong contiguous staining for platelet derived growth factor receptorβ, PDGF-Rb (B), in control brains ($n = 5$). (C,D) In DM brains ($n = 7$) claudin-5 showed less intense, more cytoplasmic localization (C) and noticeable diminution in PDGF-Rb labeling of pericytes (D). (E,F) Similar changes were found in claudin-5 (E) and PDGF-Rb in HIV-infected patients ($n = 12$). (G,H). HIV-DM and almost total disappearance of PDGF-Rb (H). Arrowheads indicate the same microvessels in double stained claudin-5 (red) and PDGF-Rb (green) panels. All images were acquired with 500 ms exposure. Original magnification: A-H x400.

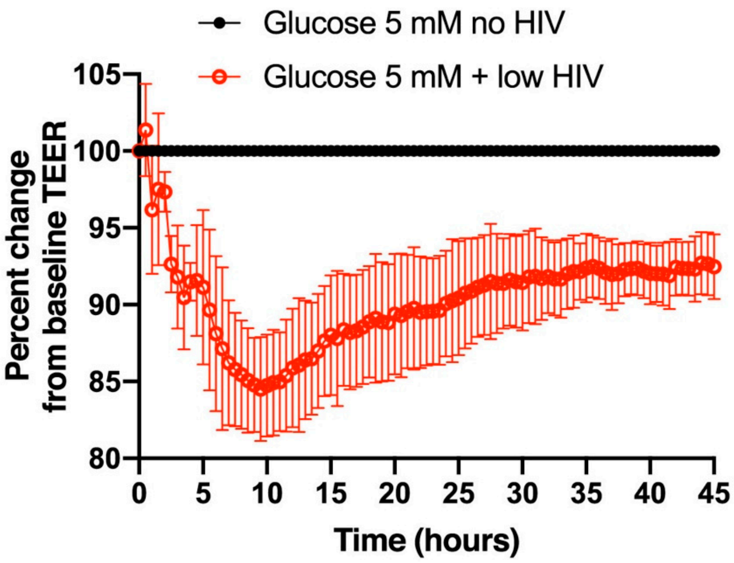

Supplemental Figure 2. *HIV exposure decreases barrier tightness.* BMVEC were maintained in medium containing 5 mM glucose until a confluent monolayer was achieved (400–600 Ohms). BMVEC were exposed to HIV at low dose. TEER was measured for 72 hr. Results are presented as percent change from baseline TEER, where 100% is defined as TEER values of non-treated (NT) cultures. Experiments were repeated twice with BMVEC from different donors and means +/- SEM of triplicate determinations are shown.
